# A case report of varicella-zoster virus-related cerebral infarction in an AIDS patient

**DOI:** 10.1097/MD.0000000000041164

**Published:** 2024-12-27

**Authors:** Rentian Cai, Fengxue Yu, Cong Cheng, Hongxia Wei

**Affiliations:** a Department of Infectious Disease, The Second Hospital of Nanjing, Affiliated to Nanjing University of Chinese Medicine, Nanjing, China; b Department of Radiotherapy, The Second Hospital of Nanjing, Affiliated to Nanjing University of Chinese Medicine, Nanjing, China.

**Keywords:** AIDS, cerebral infarction, herpes zoster, human immunodeficiency virus, meningoencephalitis, nervous system, varicella-zoster virus

## Abstract

**Rationale:**

Acquired immune deficiency syndrome (AIDS) patients with varicella-zoster virus (VZV) encephalitis rarely progress to cerebral infarction. This case highlights the potential for such complications in immunocompromised individuals.

**Patient concerns:**

A 33-year-old male with AIDS experienced recurrent hospitalizations due to neurological complications associated with VZV.

**Diagnoses:**

During the course of 3 hospitalizations, the patient’s primary diagnoses included non-herpetic herpetic neuralgia, VZV meningoencephalitis, and cerebral infarction.

**Interventions:**

The patient initially presented with herpetic neuralgia due to VZV infection. Treatment with anti-VZV acyclovir, analgesics, and anti-human immunodeficiency virus therapy improved the patient’s condition, leading to discharge. However, 8 days later, the patient developed fever, slurred speech, nausea, and vomiting. Testing confirmed VZV-related meningoencephalitis with high VZV deoxyribonucleic acid levels in the cerebrospinal fluid. Treatment included ganciclovir, foscarnet, and dexamethasone. Later, the patient suffered an acute cerebral infarction, resulting in limb paralysis and aphasia. Treatment adjustments included additional medications for VZV, human immunodeficiency virus, inflammation, antiplatelet function, and plaque stabilization, leading to improvement.

**Outcomes:**

During rehabilitation, the patient has shown improvement in right limb paralysis and aphasia, achieving grade 4 muscle strength in the affected limb and enhanced basic communication abilities. However, complete recovery has not yet occurred.

**Lessons:**

This case emphasizes the rare but significant risk of cerebral infarction in AIDS patients with VZV infection. It underscores the importance of early diagnosis, aggressive management, and continuous monitoring of neurological symptoms to mitigate long-term disability and reduce mortality in this vulnerable population.

## 1. Introduction

Varicella-zoster virus (VZV), a deoxyribonucleic acid (DNA) virus belonging to the herpesvirus family, causes primary infection resulting in chickenpox, with subsequent latency in the dorsal root and cranial nerve ganglia. Reactivation manifests as herpes zoster.^[1,2]^ VZV infection can affect the nervous system, leading to complications such as postherpetic neuralgia, meningoencephalitis, segmental sensory disturbances, facial nerve paralysis (Ramsay Hunt syndrome), spinal cord inflammation, and even cerebral infarction.^[2,3]^ These neurological complications are uncommon in immunocompetent individuals, with an incidence of 0.1% to 0.3%, but can increase to 35% in immunocompromised individuals.^[4,5]^ Cerebral infarction, as a severe complication, has been reported in several cases of immunocompetent individuals following VZV infection,^[6–8]^ but such reports are rare in populations with immunodeficiency due to human immunodeficiency virus (HIV).

We report a rare case of an acquired immune deficiency syndrome (AIDS) patient presenting with herpes zoster sine herpete complicated by extensive cerebral infarction.

## 2. Case presentation

### 2.1. Three hospital admissions

#### 2.1.1. First admission

The patient, a 33-year-old unemployed male, presented with recurring left upper abdominal pain persisting for over a month and was admitted to Nanjing Second Hospital on November 23, 2022. He reported solely left upper abdominal pain without any other discomfort. Despite multiple visits to an external hospital for treatment, the cause remained undetermined. This admission to Nanjing Second Hospital followed a positive test for HIV antibodies at the external facility, prompting further evaluation and treatment. Upon physical examination, vital signs were within normal range with a temperature of 36.6 degrees celsius (°C), heart rate of 144 beats/min, respiratory rate of 20 breaths/min, and blood pressure of 134/79 millimeters of mercury (mm Hg). The patient presented with a clear mental state and soft abdomen, exhibiting tenderness in the left upper quadrant without rebound tenderness or abnormalities elsewhere on examination. Laboratory investigations revealed a white blood cell count of 4.37 × 10^9^/L, with a neutrophil count of 2.59 × 10^9^/L; procalcitonin levels were 0.030 ng/mL, and low-density lipoprotein cholesterol was 2.5 mmol/L. Immunological assays indicated a cluster of differentiation (CD4+) T cell count of 5/microliter (μL) and a CD4+/CD8+ T cell count ratio of 0.01. Serological testing revealed positivity for VZV antibody IgG, while VZV DNA was not detected in the blood. HIV ribonucleic acid (RNA) levels were measured at 3.34 × 10^6^ copies/mL (Table 1), and Epstein-Barr virus DNA and cytomegalovirus (CMV) DNA were detected at 2.4 × 10^3^ copies/mL and 1.6 × 10^3^ copies/mL, respectively. A chest computed tomography scan with whole-abdominal enhancement revealed scattered patchy density increases in both lungs indicative of infection lesions, along with multiple tiny nodules in both lungs and small renal stones in the left kidney. The patient was diagnosed with non-herpetic herpetic neuralgia, pulmonary infection, and AIDS. Considering the medical history, the pulmonary infection was attributed to VZV. Treatment included acyclovir 250 milligrams (mg) administered every 8 hours for VZV suppression and prednisone 30 mg once daily for 5 days for anti-inflammatory purposes, resulting in significant relief of abdominal pain. On December 1st, the patient commenced a once-daily regimen of anti-HIV medication (Bictegravir 50 mg/emtricitabine 200 mg/tenofovir alafenamide 25 mg), with no adverse reactions observed. He was discharged on December 9th with continuation of anti-HIV therapy using Bituvi and prophylactic co-trimoxazole 2 tablets once daily to prevent Pneumocystis pneumonia.

**Table 1:** Peripheral blood CD4 T cell count, CD4/CD8 T cell ratio, and HIV RNA.

Number of hospitalizations	Date	CD4 T cell count (cells/μL)	CD4/CD8 cell ratio	HIV RNA (copies/mL)
The first hospitalization	November 24, 2022	5	0.01	3,340,000
The second hospitalization	December 22, 2022	98	0.06	501
	January 25, 2023	84	0.07	180
The third hospitalization	February 24, 2023	113	0.09	102

#### 2.1.2. Second admission

On December 17th, the patient presented with fever of unknown origin, accompanied by slurred speech, dizziness, headache, nausea, vomiting (non-projectile), and asymmetrical mouth corners. Despite initial treatment at an external facility, the symptoms persisted, prompting readmission to Nanjing Second Hospital on December 21st. Upon admission, vital signs were recorded as follows: temperature 36.9 °C, heart rate 115 beats/min, respiratory rate 20 breaths/min, and blood pressure 135/97 mm Hg. The patient appeared lethargic with dilated and equal-sized pupils (4mm in diameter), responsive to light reflex. Tongue extension revealed right-sided deviation, alongside skewed mouth corners to the right. The neck was supple, and muscle strength assessment revealed level 5 in both upper limbs, level 4‐ in the right lower limb, and level 4 in the left lower limb, with physiological reflexes present and no pathological reflexes elicited. Preliminary diagnosis suggested central nervous system infection, possibly AIDS.

**Figure 1.**
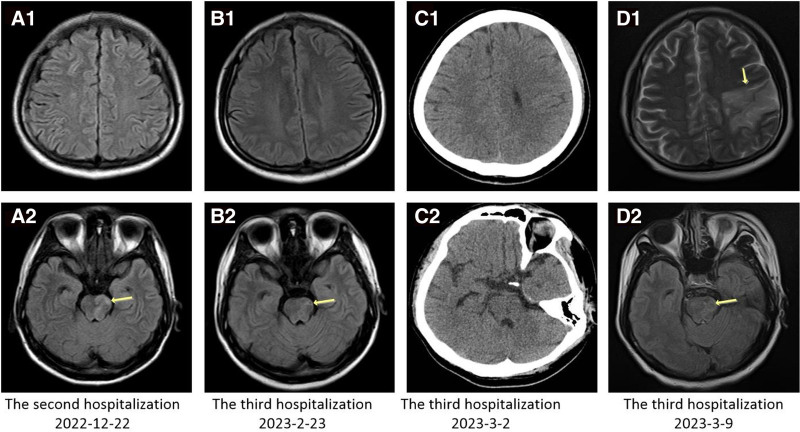
Imaging changes in the patient’s head. (A) During the second hospitalization, a brain MRI T2-Flair revealed multiple high signals in the brain stem. (B) Brain MRI T2-Flair at the beginning of the third hospitalization showed multiple high signals in the brain stem, which were slightly improved compared to those observed during the second hospitalization (Panel A). (C) The patient’s condition suddenly deteriorated during the third hospitalization, and a brain CT scan showed no apparent abnormalities. (D) Subsequent brain MRI T2-Flair during the third hospitalization showed a large area of acute cerebral infarction in the left frontal and parietal lobes, as well as multiple high signals in the brain stem. CT = computed tomography, MRI = magnetic resonance imaging.

Subsequent plain brain magnetic resonance imaging (MRI) scans displayed multiple abnormal signals in the brainstem (Fig. 1A1 and A2), while head MRA revealed no evident abnormalities. Lumbar puncture yielded cerebrospinal fluid with a pressure of 10 cm water column, showing a nucleated cell count of 43 × 10^6^/L, with mononuclear cells accounting for 100%. Cerebrospinal fluid biochemistry indicated normal levels of total protein, glucose, and lactate dehydrogenase. Next-generation sequencing technology detected VZV DNA at 44,251 copies/mL and Epstein-Barr virus DNA at 7 copies/mL in the cerebrospinal fluid, alongside HIV RNA at 1.50 × 10^2^ copies/mL (Table 2). No abnormalities were observed in cryptococcal ink staining, *Mycobacterium tuberculosis* and rifampicin resistance detection (X-pert MTB/RIF), cryptococcal capsular antigen qualitative testing, syphilis antibody testing, culture, or CMV DNA testing.

**Table 2:** Presents data on cerebrospinal fluid cells, protein, VZV DNA and HIV RNA, and blood VZV DNA.

		Cerebrospinal fluid					Blood VZV DNA
Number of hospitalizations	Date	VZV DNA	Cell count (106/L)	Percentage of mononuclear cells (%)	Total protein (mg/L)	HIV RNA (copies/mL)	
The second hospitalization	December 22, 2022	44,251	43	100	570	150	<LLD
	January 4, 2023	16,000	18	100	254	No detection	<LLD
	January 18, 2023	4800	9	89	407	No detection	No detection
	January 31, 2023	1000	10	90	444	<LLD	No detection
The third hospitalization	February 24, 2023	<LLD	29	97	1101	83	No detection
	March 3, 2023	1200	13	93	531	No detection	No detection
	March 22, 2023	<LLD	12	77	682	No detection	No detection

*Note*: The LLD of HIV RNA is 20 copies/mL; the LLD of VZV is 1000 copies/mL.

LLD = lower limit of detection.

With the revised diagnosis of VZV meningoencephalitis coexisting with AIDS, treatment involved ganciclovir 0.25 g administered intravenously via gravity drip tube every 12 hours, combined with foscarnet 3 g administered intravenously via gravity drip tube every 8 hours, and acyclovir 0.5 g administered intravenously via gravity drip tube every 8 hours for anti-VZV therapy. Additionally, dexamethasone 10 mg was administered intravenously once daily for anti-inflammatory purposes, with gradual reduction. Subsequent cerebrospinal fluid reexaminations showed a significant decrease in VZV DNA, although cell count and protein levels fluctuated (Table 2). The patient’s condition improved with normalization of body temperature, resolution of headache and vomiting, and stable muscle strength. Discharge occurred on February 3, 2023, with a prescription for continued anti-VZV therapy consisting of acyclovir 0.2 g administered orally 5 times daily and 1 tablet of anti-HIV medication once daily.

#### 2.1.3. Third hospital admission

On February 23, 2023, the patient was readmitted to Nanjing Second Hospital due to worsening slurred speech over half a day, accompanied by difficulty swallowing and episodes of choking. Upon examination, vital signs were recorded as follows: temperature 37.0 °C, heart rate 95 beats/min, respiratory rate 20 breaths/min, and blood pressure 123/87 mm Hg. The patient appeared conscious yet lethargic, with dilated pupils (4 mm in diameter), equal in size and responsive to light reflex. Tongue extension revealed right-sided deviation, alongside skewed mouth corners to the right. Muscle strength assessment indicated grade 4 ‐ in the left upper limb, grade 5 ‐ in the right upper limb, grade 2 in the right lower limb, and grade 1 in the left lower limb, with physiological reflexes present and no pathological reflexes elicited. Subsequent physical examinations yielded no positive signs.

A provisional diagnosis of possible acute stroke, VZV meningoencephalitis, and AIDS was made. Subsequent brain MRI scans revealed smaller multiple abnormal signals in the brainstem compared to previous imaging (see Fig. 1B1 and B2). Lumbar puncture yielded cerebrospinal fluid with undetectable VZV DNA and CMV DNA, alongside a decrease in HIV RNA levels to 83 copies/mL (refer to Table 2 for routine biochemistry). With increased peripheral blood CD4 T cell count and decreased HIV RNA levels (refer to Table 1), coupled with cerebrospinal fluid routine biochemistry findings indicating immune reconstitution, the revised diagnosis was viral meningoencephalitis and Immune reconstitution inflammatory response syndrome.

Treatment involved ganciclovir 0.25 intravenous gravity drip tube (IVGTT) every 12 hours combined with foscarnet 3 g IVGTT every 8 hours for antiviral therapy, along with baclofen 5 mg 3 times daily to alleviate muscle tone, and Bituvi 1 tablet once daily for anti-HIV therapy. Additionally, methylprednisolone 40 mg was administered intravenously once daily for anti-inflammatory purposes. The patient exhibited significant improvement in muscle strength and muscle tone, with recovery observed in both lower limbs.

On March 2, 2023, the patient experienced sudden aphasia accompanied by weakness, stiffness, and increased muscle tone in the right limb, along with twitching of both lower limbs. Examination revealed conscious yet lethargic presentation, aphasia, and equal-sized pupils responsive to light reflex, with muscle strength assessed at level 1 in the right upper limb and right lower limb. Pap sign was positive bilaterally. Lumbar puncture revealed VZV DNA at 1200 copies/mL, and emergency head computed tomography showed no obvious abnormalities (see Fig. 1C1 and C2). Repeat brain MRI scans revealed a large-scale acute cerebral infarction in the left fronto-parietal lobe, alongside scattered acute lacunar infarctions in the brainstem (see Fig. 1D1 and D2), with occlusion of the left posterior cerebral artery distally and localized stenosis of the right posterior cerebral artery. A diagnosis of acute cerebral infarction was established.

Treatment included administration of Edarafone 25 mg IVGTT twice daily to scavenge free radicals and protect the brain, aspirin 0.1 g once daily for antiplatelet aggregation, and atorvastatin 20 mg once nightly to lower blood lipids and stabilize plaques. Antiviral, anti-inflammatory, and anti-HIV medications were continued. Following treatment, the patient’s condition improved, and discharge from the hospital occurred on April 3, 2023, with continued oral aspirin, atorvastatin, and Bituvi therapy.

### 2.2. Main experimental methods

#### 2.2.1. Ethics statement and patient consent for publication

Ethical approval was waived by the ethics committee, as this article is a case report and all tests performed were routine examinations for the disease during that period. The written informed consent was secured from the patient.

#### 2.2.2. Main reagents

Lymphocyte subpopulation detection reagents were procured from BD Company in the United States, while VZV nucleic acid determination kits were obtained from Shanghai Zhijiang Biotechnology Co., Ltd., Shanghai, China.

#### 2.2.3. Lymphocyte subpopulation detection

The lymphocyte subpopulation detection reagents included CD3 FITC, CD4 PE-Cy7, and CD8 APC-Cy7. To conduct the test, 20 μL of lymphocyte subpopulation detection reagent was moved to the bottom of a reagent tube. Subsequently, 50 μL of thoroughly mixed anticoagulated whole blood was drawn and placed at the bottom of the test tube, ensuring no blood remained on the side wall. The test tube was covered, gently swirled to mix, and incubated in the dark at room temperature (20–25 °C) for 15 minutes. Following incubation, 450 μL of BD with a concentration of 1× for flow cytometry Cyto-Lysin was added to the tube. The tube was covered, gently shaken to mix, and incubated in the dark at room temperature (20–25 °C) for another 15 minutes. Subsequently, the sample was detected and analyzed using flow cytometry.

#### 2.2.4. VZV nucleic acid determination

##### 2.2.4.1. Blood VZV DNA determination

Two milliliter of anticoagulated blood was taken, and after allowing it to stand for natural stratification, the plasma layer was aspirated. The sample was then centrifuged at 13,000 rpm for 2 minutes, the supernatant was removed, and 50 μL of DNA extraction solution was directly added to the sediment. The mixture was thoroughly mixed and placed in a boiling water bath for 10 minutes. After centrifugation at 13,000 rpm for 5 minutes, 4 μL of the supernatant was taken for PCR reaction. The VZV nucleic acid fluorescence PCR detection mixture (35 μL), 1 μL of internal control substance, and 0.4 μL of enzyme (Taq + UNG) were combined, shaken, and centrifuged. Subsequently, 36 μL of the mixture was placed in a PCR tube, and 4 μL each of the sample processing supernatant, H_2_O, and positive control substance were added into the PCR tube. The tube was covered, centrifuged, and immediately subjected to the PCR reaction. The reaction tube was placed on a quantitative fluorescence PCR instrument, and the recommended cycle parameter setting was followed for fluorescence detection.

##### 2.2.4.2. Determination of VZV DNA in cerebrospinal fluid

Two milliliter of cerebrospinal fluid was taken and centrifuged at 13,000 rpm for 2 minutes. The supernatant was removed, and 50 μL of DNA extraction solution was directly added to the sediment. After thorough mixing, the mixture was bathed in a boiling water bath for 10 minutes. Following centrifugation at 13,000 rpm for 5 minutes, 4 μL of the supernatant was taken for PCR reaction, and subsequent steps were similar to those described above.

## 3. Differential diagnosis

The differential diagnoses for the AIDS patient presenting with varicella-zoster virus-related cerebral infarction, accompanied by right limb paralysis and aphasia, include brain tumors, brain abscesses, and cerebral hemorrhage. A shorter duration of symptoms can help exclude brain tumors. MRI and cerebrospinal fluid examination are crucial for distinguishing between brain abscesses, cerebral hemorrhage, and cerebral infarction.

## 4. Outcome and follow-up

At the time of discharge, the patient presented with right limb paralysis (hemiparesis) and aphasia (a language disorder) following a neurological event, such as a stroke. The rehabilitation plan was initiated to address both motor and language impairments, with the goal of maximizing functional recovery. The patient has been undergoing structured rehabilitation exercises aimed at restoring motor function in the right limb. Muscle strength has improved significantly, progressing to grade 4 on the Medical Research Council (MRC) scale. This indicates good muscle strength (the patient can move the affected limb against gravity and moderate resistance), although it is not yet at full strength (grade 5). The patient’s aphasia has shown notable improvement, with recovery to the level of basic communication. The patient can engage in simple conversations, express needs, and understand basic spoken language. However, fluent speech and complex language comprehension are still limited.

## 5. Discussion

The report describes a case of an AIDS patient with severe immunodeficiency who developed acute cerebral infarction following VZV infection. The patient exhibited risk factors for cerebral infarction, including confirmed VZV meningoencephalitis and HIV infection, both of which likely contributed to the occurrence of cerebral infarction. Previous reports have indicated that in immunocompetent patients, VZV infection of the central nervous system can lead to meningoencephalitis, and in severe cases, cerebral infarction or hemorrhage due to VZV-induced vasculitis,^[6,7]^ attributed to vascular inflammation caused by VZV.^[9]^ A previous case report of a 14-year-old male showed inflammatory cell infiltration, vascular sheath-like changes, and fibrinoid necrosis on brain biopsy following VZV infection leading to cerebral infarction.^[8]^ While no brain biopsy was performed in our case, the presence of VZV in the cerebrospinal fluid and sudden stenosis of cerebral blood vessels leading to cerebral ischemic necrosis indicate an acute onset rather than gradual stenosis due to elevated cholesterol levels. Additionally, HIV, besides severely compromising immune function, also causes chronic inflammation, an independent risk factor for cardiovascular and cerebrovascular diseases, albeit over a chronic course.^[10–12]^ Therefore, the primary cause of acute cerebral infarction in this patient is presumed to be VZV infection of the nervous system.

VZV-induced vascular inflammation can result from both viral factors and immune-mediated mechanisms. In cases of compromised immunity, severe vascular inflammation may not develop. The use of anti-HIV medications effectively suppresses HIV and promotes immune reconstitution, potentially triggering VZV-induced vascular inflammation. Recently, a case report described a 55-year-old male with confirmed VZV infection leading to cerebral infarction. Treatment with acyclovir for VZV, ritonavir for HIV, and methylprednisolone to reduce inflammatory responses resulted in decreased VZV levels in cerebrospinal fluid below detectable limits, significant reductions in peripheral blood HIV RNA, and marked increases in CD4 T cell count. However, repeat brain MRI showed progression of cerebral infarction lesions, indicating that immune recovery exacerbated VZV-related cerebral infarction.^[13]^ Similar to this case, our patient experienced VZV-related cerebral infarction during immune recovery. Currently, there is no established treatment protocol for VZV-related cerebral infarction. Literature reviews suggest consensus on aggressive anti-VZV treatment, but opinions vary regarding aspirin for antiplatelet therapy, heparin for anticoagulation, and steroids for anti-inflammatory effects, with different treatment approaches reported in individual cases.^[7,14–17]^ The case we report here is particularly complex: despite aggressive anti-VZV, anti-inflammatory, and anti-HIV therapies, the patient still experienced extensive cerebral infarction. Subsequent aggressive antiplatelet therapy with aspirin and statin stabilized the plaques, leading to clinical stabilization and functional recovery of language and limb functions after rehabilitation exercises.

In HIV-infected individuals, latent VZV activity may complicate neurological diseases, including cerebral infarction. Therefore, further research is needed to determine whether a robust anti-HIV regimen aimed at rapidly restoring immune function or a more moderate regimen aimed at reducing the risk of immune reconstitution can prevent serious cerebrovascular accidents in such patients.

## 6. Learning points

Cerebral infarction in AIDS patients with VZV infection of the nervous system is uncommon but can occur, indicating the need for heightened awareness and vigilance.

The case underscores the importance of comprehensive and multidisciplinary management in such complex cases to mitigate mortality and disability. Vigilant monitoring of neurological symptoms is crucial for timely intervention.

Further research is needed to better understand the mechanisms linking AIDS, VZV infection, and cerebral infarction. This includes refining treatment protocols and exploring preventive strategies to improve outcomes in affected patients.

## 7. Limitations

This case report is limited by its nature as a single case study, which may limit the generalizability of the findings. The patient’s outcomes are specific to his individual clinical course and may not represent typical results in other patients with similar conditions. Additionally, the role of VZV-induced cerebral infarction in immunocompromised patients remains under-researched, and additional studies with larger samples and longer follow-up are necessary to fully understand the mechanisms and treatment options for this complication.

## Acknowledgments

We are grateful to the patient for allowing us to document their medical history.

## Author contributions

**Conceptualization:** Rentian Cai, Hongxia Wei.

**Data curation:** Rentian Cai, Hongxia Wei.

**Formal analysis:** Fengxue Yu.

**Investigation:** Hongxia Wei.

**Methodology:** Rentian Cai.

**Software:** Fengxue Yu.

**Writing – original draft:** Rentian Cai.

**Writing – review & editing:** Cong Cheng, Hongxia Wei.
